# Long-Term Management of Esophageal Varices by Endoscopic Sclerotherapy (EST): A Review of 12 Years' Experience

**DOI:** 10.1155/DTE.2.211

**Published:** 1996

**Authors:** Dinesh K. Bhargava, S. Dasarathy, Sushma Saksena

**Affiliations:** 1 Department of Gastroenterology All India Institute of Medical Sciences New Delhi 110029 India

## Abstract

A total of 566 patients with variceal bleeding caused by cirrhosis of the liver, noncirrhotic portal fibrosis (NCPF) and extrahepatic portal venous obstruction (EHO) were treated by repeated endoscopic injection sclerotherapy. This decreased rebleeding was evidenced by a reduction in mean bleeding risk factor and transfusion requirement. Both the factors were significantly (*P* < 0.001) decreased in all three groups of patients. Rebleeding occurred before eradication in 27.7% of patients with cirrhosis, 24.3% of those with NCPF, and 11% of those with EHO. Significantly more patients with cirrhosis and NCPF bled in comparison to EHO. Irrespective of the etiology, fewer patients of Child's A class bled than those of Child's B and C classes (*P* < 0.001). The median bleeding-free period was longer in patients with EHO than in those with cirrhosis (*P* < 0.05). This period was also significantly longer in Child's A class than in Child's B and the latter had a longer median bleeding-free period than Child's C class (*P* < 0.01). Variceal eradication was achieved in 80% of patients with cirrhosis, 87% of patients with NCPF, and 90% of patients with EHO. The success of variceal eradication was higher in EHO patients in contrast with patients with cirrhosis of the liver. Similarly, eradication was better in Child's A class patients than in Child's B and C class patients. Recurrence of varices and complications were not influenced by the Child's status or etiology of portal hypertension. The probability of survival at 10 years was higher in patients with EHO (88%) and NCPF (80%) than in patients with cirrhosis (50%). Similarly, patients with Child's A (88%) status survived longer than those with Child's B (42%) status, and patients with Child's B status had a longer survival than Child's C status patients (0%). Thus, endoscopic variceal sclerotherapy appears to be a useful procedure for the long-term management of patients after an esophageal variceal bleeding irrespective of the etiology of portal hypertension.


*Diagnostic and Therapeutic Endoscopy*, 1996, Vol. 2, pp. 211–217
Reprints available directly from the publisher
Photocopying permitted by license only

© 1996 OPA (Overseas Publishers Association)
Amsterdam B. V. Published in The Netherlands
by Harwood Academic Publishers GmbH
Printed in Singapore

### Long-Term Management of Esophageal Varices by Endoscopic Sclerotherapy (EST): A Review of 12 Years' Experience

DINESH K. BHARGAVA, S. DASARATHY, and SUSHMA SAKSENA

*Department of Gastroenterology, All India Institute of Medical Sciences, New Delhi, India*

*(Received June 5, 1995; in final form November 13, 1995)*

A total of 566 patients with variceal bleeding caused by cirrhosis of the liver, noncirrhotic portal fibrosis (NCPF) and extrahepatic portal venous obstruction (EHO) were treated by repeated endoscopic injection sclerotherapy. This decreased rebleeding was evidenced by a reduction in mean bleeding risk factor and transfusion requirement. Both the factors were significantly ($P < 0.001$) decreased in all three groups of patients. Rebleeding occurred before eradication in 27.7% of patients with cirrhosis, 24.3% of those with NCPF, and 11% of those with EHO. Significantly more patients with cirrhosis and NCPF bled in comparison to EHO. Irrespective of the etiology, fewer patients of Child's A class bled than those of Child's B and C classes ($P < 0.001$). The median bleeding-free period was longer in patients with EHO than in those with cirrhosis ($P < 0.05$). This period was also significantly longer in Child's A class than in Child's B and the latter had a longer median bleeding-free period than Child's C class ($P < 0.01$). Variceal eradication was achieved in 80% of patients with cirrhosis, 87% of patients with NCPF, and 90% of patients with EHO. The success of variceal eradication was higher in EHO patients in contrast with patients with cirrhosis of the liver. Similarly, eradication was better in Child's A class patients than in Child's B and C class patients. Recurrence of varices and complications were not influenced by the Child's status or etiology of portal hypertension. The probability of survival at 10 years was higher in patients with EHO (88%) and NCPF (80%) than in patients with cirrhosis (50%). Similarly, patients with Child's A (88%) status survived longer than those with Child's B (42%) status, and patients with Child's B status had a longer survival than Child's C status patients (0%). Thus, endoscopic variceal sclerotherapy appears to be a useful procedure for the long-term management of patients after an esophageal variceal bleeding irrespective of the etiology of portal hypertension.

**KEY WORDS:** Endoscopic sclerotherapy for esophageal varices, cirrhosis of the liver, noncirrhotic portal fibrosis, extrahepatic portal venous obstruction

### INTRODUCTION

Since 1981, we have been injecting esophageal varices during active variceal bleeding and for long-term management after variceal bleeding (1–4). In India, besides cirrhosis of the liver, two other diseases, noncirrhotic portal fibrosis (NCPF), and extrahepatic portal venous obstruction (EHO) are also responsible for portal hypertension (5).

Between 1981 and 1993, we treated 566 patients with portal hypertension, who had bleeding from esophageal varices. The varices were caused by cirrhosis of the liver, NCPF, and EHO. These are few studies on the long-term results of the use of endoscopic sclerotherapy (EST) for the management of patients who had bled from esophagastic varices as in the present study. The efficacy of this mode of treatment is retrospectively reviewed. This study also compares the results among the three groups of patients. This is one of the largest groups of patients reported with a long and complete follow-up.

### METHODS

Between January 1981 and June 1993, 566 consecutive patients with portal hypertension underwent EST of esophageal varices: 342 with cirrhosis of the liver, 107

Address for correspondence: Dr. D.K. Bhargava, Professor of Gastroenterology, All India Institute of Medical Sciences, New Delhi-110029, India.

with NCPF and 117 with EHO (Table 1). The etiology of cirrhosis was alcohol-related in 119, posthepatic in 91, and cryptogenic in 132 patients. Informed consent was obtained from all patients. All of them were seen with endoscopically documented variceal hemorrhage requiring blood transfusion in the recent past (< 30 days).

In each patient endoscopy confirmed grade 3 or 4 esophageal varices (6). All patients had a detailed clinical evaluation. Hepatic cirrhosis was verified by liver biopsy or considered the most likely diagnosis on the basis of clinical, biochemical, and imaging criteria. The diagnosis of NCPF was established by the exclusion of cirrhosis of the liver on histologic examination and EHO. The diagnosis of EHO was made on ultrasound and/or splenoportovenogram. The hepatic functional reserve was classified by the Pugh's modification of the Child's scoring system (7). Variceal rebleeding was defined by a definite history of hematemesis and melena with endoscopic evidence of active bleeding or the presence of varices with no other lesion in the upper gastrointestinal tract (1).

### Technique

Sclerotherapy was carried out using an Olympus Q10, XQ10, XQ20, or video forward-viewing fiberoptic panendoscope and NM-1K injector. Polidocanol 1% solution (Aethoxysklerol, Krussler & Co., Germany) was used as a sclerosing agent. The technique of injection has already been described in detail (1). The injections were repeated every 3 weeks until July 1991. Subsequently, the varices were injected weekly. After eradication, endoscopic examination was repeated every 3 to 6 months to observe any recurrence of varices. The new varices were injected in a similar manner until eradication was achieved.

### Statistical Methods

The qualitative variables were analyzed using the $\chi^2$ test. Quantitative variables were analyzed by Student's *t* test and those with skewed values by Wilcoxon's rank-sum test. The Kaplan-Meier method was used to estimate the probabilities of survival and bleeding-free period. The resulting curves were compared using the log-rank test.

**Table 1** Patient characteristics.

| | *Cirrhosis* | *NCPF* | *EHO* |
|---|---|---|---|
| No. | 342 | 107 | 117 |
| M/F | 302/40 | 77/30 | 76/41 |
| Age(yr.) (mean ± SD) | 43.6 ± 19.8 | 29.8 ± 14.6 | 15.1 ± 8.9 |
| Child's score | | | |
| A | 114 | 90 | 117 |
| B | 180 | 17 | |
| C | 48 | | |

### RESULTS

Before the initiation of EST, the episodes of bleeding and blood transfusion requirements were similar in the patients with cirrhosis, NCPF, and EHO ($P > 0.1$). Rebleeding before and after EST was compared using 1) mean bleeding risk factor (BRF), defined as the number of rebleeding episodes per patient per month of follow-up; and 2) mean transfusion requirements. Both were significantlty ($P < 0.001$) decreased in all three groups of patients after EST (Table 2). Patients with cirrhosis of the liver, NCPF, and EHO were followed for a mean period of 64, 72, and 73 months respectively. Nineteen percent of patients were lost to follow-up during the study period. Patients lost to follow-up were analyzed up to the point they were last seen.

### Rebleeding Parameters

#### *Patients Rebleeding*

The numbers of patients whose varices rebled between the first session of EST and eradication in the different etiological subgroups and stratified for Child's scores are given in Table 3. Significantly more patients with cirrhosis had rebleeding (27.7%) compared with those with EHO ($P < 0.0001$). The number of patients with NCPF who had rebleeding (24.3%) was significantly more ($P < 0.05$) than those with EHO (11.1%). There was no difference in the rebleeding rates between patients with cirrhosis and NCPF ($P > 0.1$).

**Table 2** Comparison of Rebleeding Parameters

| | *Mean BRF* | | | *Mean Transfusion* | | |
|---|---|---|---|---|---|---|
| | *Before EST* | *After EST* | *P Value* | *Before EST* | *After EST* | *P Value* |
| Cirrhosis | 0.41 | 0.027 | <0.001 | 6.4 ± 6.1 | 2. ± 1.6 | <0.001 |
| NCPF | 0.16 | 0.008 | <0.001 | 7.1 ± 5.9 | 1.3 ± 2.3 | <0.001 |
| EHO | 0.12 | 0.005 | <0.001 | 6.1 ± 7.3 | 1.2 ± 2.2 | <0.001 |

BRF: Bleeding risk factor; EST: Endoscopic sclerotherapy

**Table 3** Number of Patients and Episodes of Rebleeding

| *Etiology* | *A* | *B* | *C* | *Total* |
|---|---|---|---|---|
| Cirrhosis | 24 | 46 | 25 | 95 (27.7%) |
| | (30) | (51) | (48) | (129) |
| NCPF | 17 | 9 | 0 | 26 (24.3%) |
| | (28) | (22) | | (50) |
| EHO | 13 | 0 | 0 | 13 (11.1%) |
| | (18) | | | (18) |
| Total | 54 (16.8%) | 55 (27.9%) | 25 (52.08%) | |

Figures in parentheses indicate number of episodes of rebleeding.

Analysis of the influence of Child's status irrespective of etiology showed that significantly fewer patients of Child's A class (16.8%) bled than those of Child's B class (27.9%) or Child's C class (52.08%) while patients of Child's B status bled less than those of Child's C status ($P < 0.001$).

### *Bleeding-Free Period*

The probability of the bleeding-free period (BFP) was calculated by the Kaplan-Meier method. This was significantly longer (Fig. 1) in patients with EHO (>120 months) than in those with cirrhosis ($P < 0.05$). There was no significant difference in the median BFP ($P > 0.1$) between patients with cirrhosis (> 120 months) and NCPF (> 120 months). The etiology of portal hypertension did not affect the BFP for a given Child's class of patients. Patients of Child's A status (Fig. 2) irrespective of etiology had a longer BFP (> 19 months) in contrast to Child's B (> 98 months) or C (> 12 months) patients. Similarly, patients in Child's B class had a longer BFP than those in Child's C class ($P < 0.01$).

### Variceal Eradication and Recurrence

Variceal eradication was achieved in 274 patients with cirrhosis (80%), 79 patients with NCPF (87%), and 105 patients with EHO (90%). The success of eradication was significantly higher in patients with EHO than in those with cirrhotics ($P < 0.05$). This was also related to the Child's status of patients, as eradication occurred in 88.8% of patients having a Child's A status and 76.3% among the Child's B and C group. There was no difference among patients having similar Child's status within different etiologic subgroups ($P > 0.05$). The mean number of sessions of EST required for eradication was 7.9 (±3.8) in patients with cirrhosis, 7.6 (±3.2) in those with NCPF, and 7.4 (±3.6) in those with EHO and were similar ($P > 0.1$).

Recurrence of esophageal varices was observed in 21% of patients and was not affected by the etiology of portal hypertension or the Child's score ($P > 0.05$). Of these, 21% of patients with recurrence of varices, 26% were seen with variceal bleeding. Recurrences were observed after a mean period of 38.9 ± 22.6 months and were between grade 1 and grade 2 at the onset. These were reinjected by the same technique until eradication (mean 1.3 sessions).

### Causes of Death

There were 89 (26%) deaths among patients with cirrhosis of the liver. Seven (6.5%) patients with NCPF and 2 (1.70%) patients with EHO died on follow-up. In patients with cirrhosis of the liver, hepatic encephalopathy was the direct major cause of death in 65 patients, uncontrolled variceal bleeding in 15, spontaneous bacterial peritonitis in 2, hepatorenal syndrome in 3, septicemia in 3, and hepatocellular carcinoma in 1. Among patients with NCPF, four died of a combination of variceal hemorrhage and hepatic coma. The remaining patients with NCPF and EHO died of exsanguinating hemorrhage. There were 8 (7%) of Child's A, 51 (28.33%) of Child's B, and 30 (62.5%) of Child's C class patients with cirrhosis who died. There were two deaths (2.2%) among patients with NCPF of Child's A class and 5 (29.41%) of Child's B class. The mortality was significantly lower ($P < 0.01$) in patients of Child's A class than in patients of Child's B class and lower in Child's B than C class ($P < 0.01$) patients, irrespective of etiology.

### Survival

An analysis of the survival by the Kaplan-Meier technique showed that the probability of survival at 10 years was 50, 80, and 88% in patients with cirrhosis, NCPF, and EHO, respectively (Fig. 3). Patients with NCPF and EHO survived longer than patients with cirrhosis ($P < 0.01$). There was no statistical differences between patients with NCPF and EHO ($P > 0.05$). The probability of survival at 10 years was 88% for Child's A, 42% Child's B, and 0% for Child's C class patients (Fig. 4). Patients belonging to Child's A status irrespective of etiology had significantly ($P < 0.001$) longer survival than those of Child's B or C class. Similarly patients of Child's B status survived longer than those of Child's C status ($P < 0.01$).

### Complications

Complications such as retrosternal pain, fever of > 100°F, deep ulcers, dysphagia, stricture, mediastinitis, pneumonia, and pleural effusions were observed in 15% of our patients after EST. The Child's status and the etiology of portal hypertension did not influence the incidence of complications significantly. Mucosal slough or local superficial ulcers were excluded from the list of complications as they were usually of little significance in the overall management of the patients.

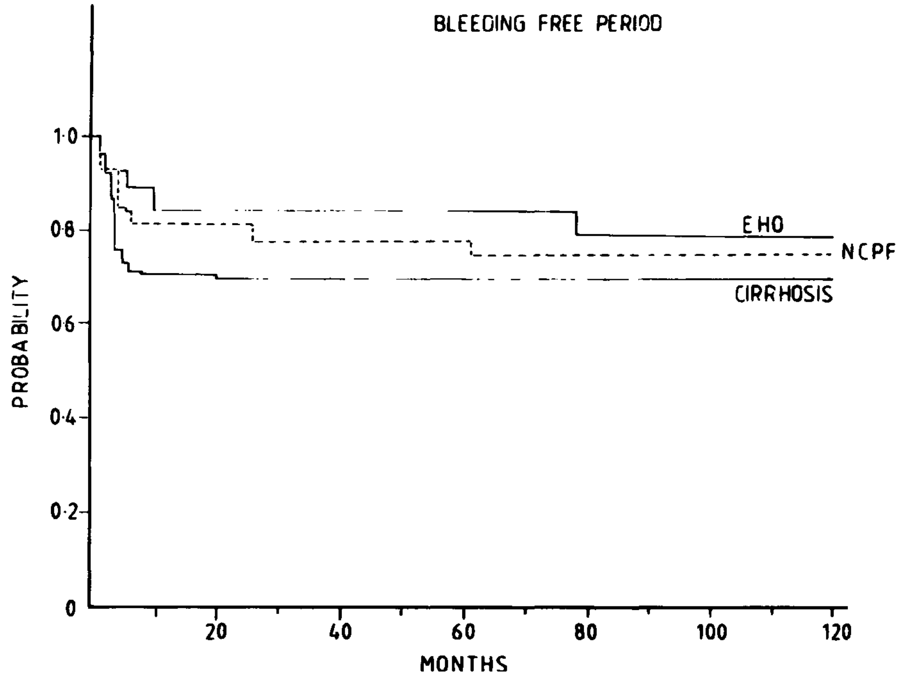

**Figure 1** Bleeding-free period after EST: this was significantly longer in patients with EHO than in those with cirrhosis of the liver.

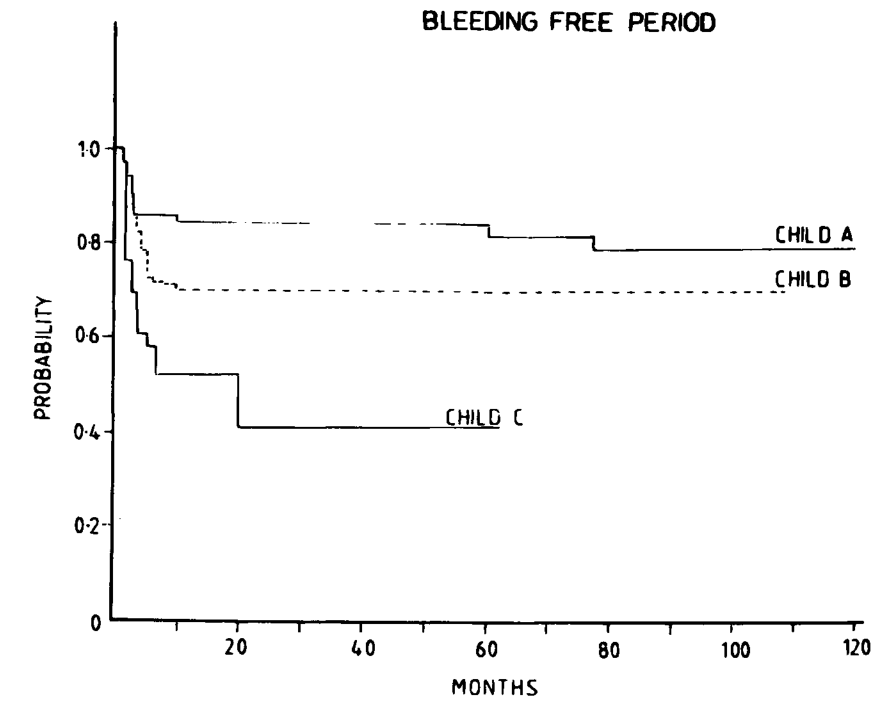

**Figure 2** Bleeding-free period after EST: patients of Child's A status had a longer bleeding-free period compared with Child's B or C status patients and patients of Child's B class had longer than patients with Child's C class.

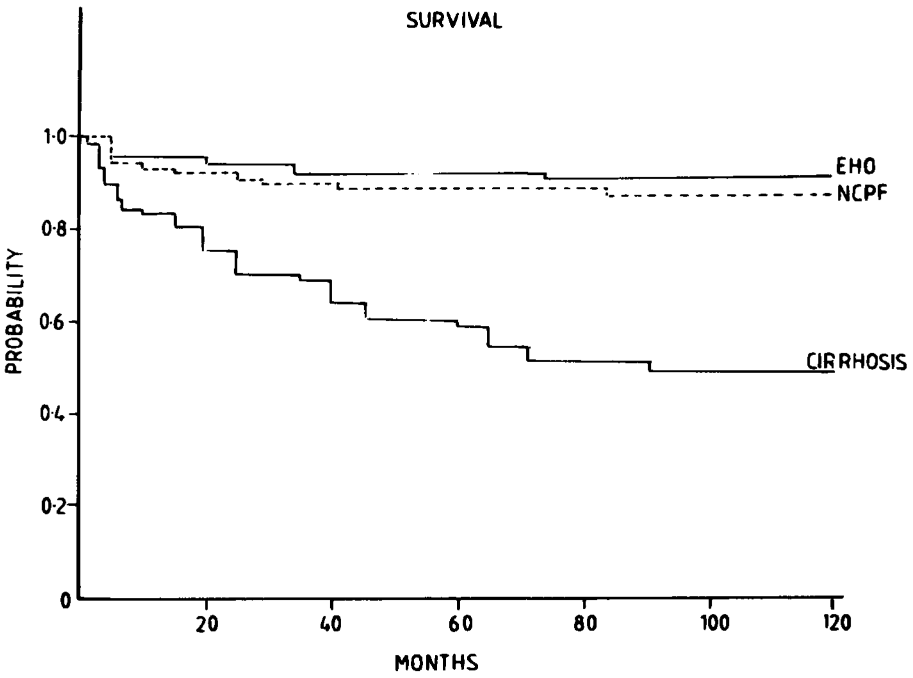

**Figure 3** Cumulative survival rates after sclerotherapy by life for patients with EHO, NCPF, and cirrhosis. Patients with EHO and NCPF survived longer than those with cirrhosis.

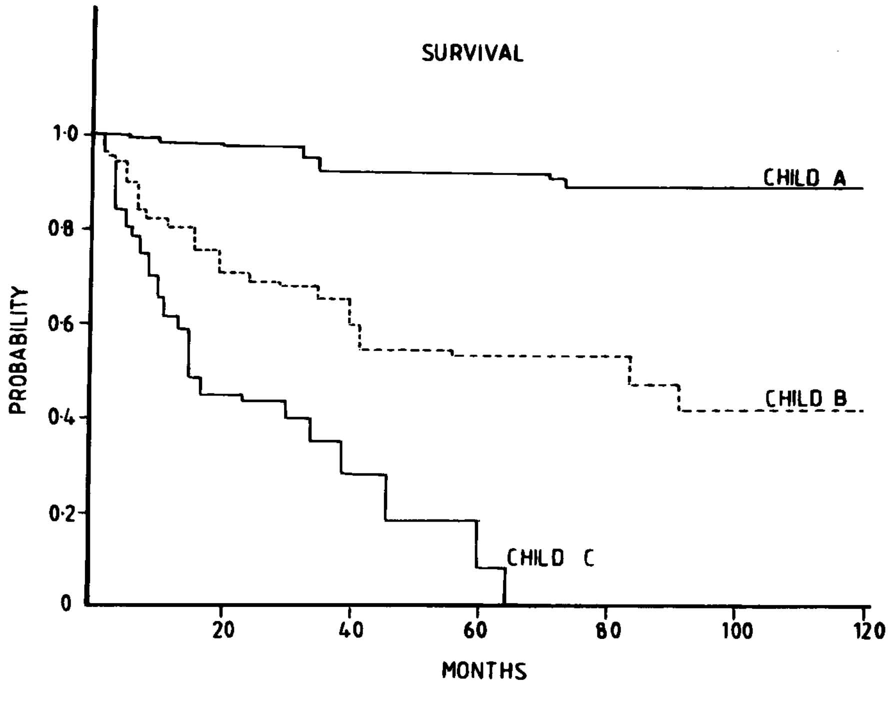

**Figure 4** Cumulative survival rates after sclerotherapy for patients according to their Child's status. Patients of Child's A class survived longer than those of Child's C class. Survival was longer in patients of Child's B class than those of Child's C class.

### DISCUSSION

Repeated sclerotherapy has been found to be a safe and effective mode of treatment for the long-term management of esophageal variceal bleeding irrespective of the etiology of portal hypertension. This is supported by controlled and prospective trials (1–3,8,9). The place of long-term β-blockade remains controversial (10). Surgical procedures have significant morbidity and mortality.

One of the major disadvantages of repeated EST was rebleeding episodes before eradication of varices. These occurred more often in patients of Child's C status than in those of Child's B class and more in Child's B status than Child's A status patients in the present study. Similarly, patients with better hepatic function had a significantly longer BFP. In terms of etiology, results of rebleeding parameters favored patients with EHO more than those with NCPF and cirrhosis of the liver. This was supported by the observation that there was no significant difference between different etiologic categories within each Child's class. Similar observations have been made by Sakai et al. (11) reported better results in patients with schistosomiasis than in those with cirrhosis.

It was observed that rebleeding episodes were insignificant once the varices are eradicated. This was achieved in 80% of patients with cirrhosis, 87% of patients with NCPF, and 90% of patients with EHO. The mean number of sclerotherapy sessions required for eradication were comparable in the three groups. Eradication rate was better achieved in Child's A class patients than in Child's B and Child's C class patients ($P < 0.05$). It was difficult to eradicate varices in the remaining patients, and in many of varices continue to bleed intermittently. These should be considered as sclerotherapy failures. Such patients should probably be subjected to an alternate procedure such as surgery or endoscopic band ligation. Terblanche et al. (9) also reported that 10% of their patients failed to respond to sclerotherapy. The recurrence of varices was observed in 21% of patients after a mean period of 38.9 ± 22.6 months irrespective of etiology or Child's status. The lower incidence of recurrence in our series was possibly due to total eradication of varices at the begining of treatment and a regular follow-up of patients at an interval of 3 to 6 months. Thus, prevention of recurrent bleeding was dependent upon the successful eradication of varices, and regular surveillance is necessary to ensure eradication.

It is not yet clear whether repeated sclerotherapy improves survival. Meta analysis of the data from controlled trials did, however, show a survival advantage for sclerotherapy (12). The overall cumulative survival rate at 10 years was 50, 80, and 88% in patients with cirrhosis of the liver, NCPF, and EHO, respectively. The cumulative 10-year survival rate was 88% for Child's A, 42% for Child's B, and 0% for Child's C class patients. As expected Child's status of the patients did influence the survival. The etiology of the patients also influenced survival as patients with EHO and NCPF survived longer than those with cirrhosis. This could be because the majority of patients with NCPF and EHO belonged to Child's A status. This observation was similar to that of others (9,11) and has also been emphasized by us in the past (1).

Usually, complications related to the procedure were minor except for esophageal stricture formation, mediastinitis, pleural effusion, and pneumonia. Superficial ulcers and mucosal slough were of little significance as they were only encountered during repeated endoscopy. It is suggested that this leads to better eradication of varices and control of recurrent bleeding episodes (13,14). Complications also vary with the type of sclerosant (15,16) used as well as the quantity, concentration, and frequency of injections (17).

Thus, the present study confirms that repeated endoscopic sclerotherapy prevents rebleeding by eradication of varices. The results were influenced primarily by the functional hepatic status and indirectly by the etiology of portal hypertension. Eradication is most successful in patients with EHO, followed by NCPF and cirrhosis of the liver. It is difficult to comment whether eradication of varices increases the survival. However, at least patients with EHO and NCPF have an excellent survival rate. On the basis of our observations, repeated endoscopic sclerotherapy is the procedure of choice for the long-term management of esophageal varices at our center, irrespective of the etiology of portal hypertension and their functional status. We are injecting varices every week with 1% polidocanol until they are eradicated. In case of failure of sclerotherapy, we resort to either endoscopic band ligation or surgery. Once the varices are eradicated, endoscopy is performed at an interval of 3 to 6 months to detect early recurrence of varices. Recurrent varices should be treated similarly.

### REFERENCES

1. Bhargava DK, Dasarathy S, Sundaram KR, et al. Efficacy of endoscopic sclerotherapy on long term management of esophageal varices: a comparative study of results in patients with cirrhosis of the liver, non-cirrhotic portal fibrosis and extrahepatic portal venous obstruction. J Gastroenterol Hepatol 1991;6:471–475.
2. Bhargava DK, Dwivedi M, Dasarathy S, et al. Sclerotherapy after variceal hemorrhage in non-cirrhotic portal fibrosis. Am J Gastroenterol 1989;84:1235–1238.
3. Bhargava DK, Dwivedi M, Dasarathy S, et al. Endoscopic sclerotherapy for portal hypertension due to extrahepatic obstruction: results and long term follow-up. Gastrointest Endosc 1989;35:309–311.

4. Bhargava DK, Dasarathy S, Atmakuri SP, et al. Comparative efficacy of emergency endoscopic sclerotherapy for active variceal bleeding due to cirrhosis of the liver, non-cirrhotic portal fibrosis and extrahepatic portal venous obstruction. J Gastroenterol Hepatol 1990;5:432–437.
5. Anand CS, Tandon BN, Nundy S. The causes, management and outcome of upper gastrointestinal haemorrhage in an Indian Hospital. Br J Surg 1983;70:209–211.
6. Conn HO. Ammonia tolerance in the diagnosis of esophageal varices: a comparison of endoscopic, radiological and biochemical techniques. J Lab Clin Med 1967;70:442–451.
7. Pugh RNH, Murray-Lyon IM, Dawson JL, et al. Transection of the oesophagus for bleeding esophageal varices. Br J Surg 1973;60:646–649.
8. Terblanche J, Burrough AK, Hobbs KEF. Controversies in the management of bleeding esophageal varices. N Engl J Med 1989;320:1393.
9. Terblanche J, Kahn D, Bormann PC. Long term injection sclerotherapy treatment for esophageal varices: a 10 years prospective evaluation. Ann Surg 1989;210:725–731.
10. Dasarathy S, Dwivedi M, Bhargava DK, et al. A prospective randomised trial comparing repeated endoscopic sclerotherapy vs propranolol in poor risk cirrhotic patients. Hepatology 1992;16:89–94.
11. Sakai P, Bonaventura S, Capacci ML, et al. Endoscopic sclerotherapy of bleeding esophageal varices. A comparative study of results in patients with schistosomiasis and cirrhosis. Endoscopy 1988;20:134–136.
12. Infante-Rivard C, Esnaola S, Villeneuve JP. Role of endoscopic variceal sclerotherapy in the long term management of variceal bleeding: A meta-analysis. Gastroenterology 1989;96:1087–1092.
13. Kahn D, Jones B, Bornman PC, et al. Incidence and management of complications after injection sclerotherapy: A 10 years prospective evaluation. Surgery 1989;105:160–165.
14. Kitano S, Koyanagi N, Iso Y, et al. Prevention of recurrence of esophageal varices after endoscopic injection sclerotherapy with ethanolamine oleate. Hepatology 1987;7:810–815.
15. Bhargava DK, Atmakuri SP, Sharma MP. Endoscopic sclerotherapy for esophageal varices using absolute alcohol. Gut 1986;27:1518.
16. Bhargava DK, Singh B, Dogra R, et al. Prospective randomized comparison of sodium tetradecyl sulfate and polidocanol as variceal sclerosing agents. Am J Gastroenterol 1992;87:182–186.
17. Sorensen TIA, Burcharth F, Federson ML, et al. Esophageal stricture and dysphagia after endoscopic sclerotherapy for bleeding varices. Gut 1984;25:473–477.

